# Model Animal Mimicking Human Virus-induced Diabetes

**DOI:** 10.1016/j.ebiom.2018.05.018

**Published:** 2018-05-19

**Authors:** Keiichiro Mine, Hirokazu Takahashi, Seiho Nagafuchi

**Affiliations:** a Division of Host Defense, Medical Institute of Bioregulation, Kyushu University, 3-1-1, Maidashi, Higashi-ku, Fukuoka 812-8582, Japan; b Division of Metabolism and Endocrinology, Department of Internal Medicine, Faculty of Medicine, Saga University, 5-1-1, Nabeshima, Saga 849-8501, Japan

Infectious agents, particularly *Coxsackievirus B* (CVB) group belonging to the *Enterovirus* genus of the family *Picornaviridae*, have been suggested to be the cause of virus-induced type 1 diabetes (T1D). Recently, Stone *et al*. showed that a CVB1 vaccine can protect CVB1-induced diabetes in suppressor of cytokine signaling 1 (SOCS1)-transgenic NOD mice [1]. It has been reported that inoculation of antiserum against diabetogenic D variant of murine encephalomyocarditis (EMC-D) virus prevented diabetes, only when transferred within 36 h after infection. These studies suggested that antiserum treatment at an early stage of virus-induced T1D is not feasible and therefore vaccination against diabetogenic viruses to prevent T1D is essentially required.

Even diabetogenic EMC-D virus could induce overt diabetes only in limited mice strains, indicating that the combination of viral diabetogenicity and host genetics is essential to reach clinical diabetes. In addition, several strains exhibit moderate susceptibility to EMC-D, implying that minor susceptibility gene(s) contribute to the etiology.

Hober and Alidjinou claimed that a multivalent vaccine for prevention of T1D could be designed to include all CVBs serotypes, and also claimed that the precise composition of multivalent vaccine is important [2]. Although assessment of viral diabetogenicity is essential for developing a precisely composed vaccine, diabetogenic and/or latently diabetogenic viruses have not yet been clearly defined.

Several studies have showed that animals with human disease risk variant(s) help us to understand the etiologic mechanisms of human diseases. Based on *Tyrosine kinase 2* (*Tyk2*) gene as a murine EMC-D virus-induced diabetes susceptibility gene [3], we could show that polymorphisms in 5′UTR of *TYK2*, named *TYK2* promoter variant (ClinVar ID: 440728), is associated with increased risk of human T1D, most highly, among flu-like syndrome associated anti-GAD antibody negatives, indicating that this variant is likely to be a human virus-induced diabetes susceptibility gene [4,5]. Since virus-induced diabetes is dependent on the combination risk as diabetogenictity of the virus and susceptibility of the host, to establish animal models correctly mimicking human virus-induced diabetes, the application of *TYK2* promoter variant and/or other major/minor human virus-induced diabetes-associated genetic variants to animals is imperative. Thus, the development of these animal models harboring virus-induced diabetes susceptibility gene(s), to detect the diabetogenic viruses at a high sensitivity, will help us not only to understand the mechanisms of human virus-induced diabetes but also to identify diabetogenic and/or latently diabetogenic viruses. Such studies will contribute to the development of precise composed effective vaccines to eradicate virus-induced T1D.
